# Detection of Coronaviruses in Bats of Various Species in Italy

**DOI:** 10.3390/v5112679

**Published:** 2013-10-31

**Authors:** Davide Lelli, Alice Papetti, Cristiano Sabelli, Enrica Rosti, Ana Moreno, Maria B. Boniotti

**Affiliations:** 1 Istituto Zooprofilattico Sperimentale della Lombardia e dell’Emilia Romagna, Via Bianchi 9, Brescia 25124, Italy; E-Mails: alice.papetti@izsler.it (A.P.); anamaria.morenomartin@izsler.it (A.M.); mariabeatrice.boniotti@izsler.it (M.B.B.); 2 IZO s.r.l, Via S. Zeno 99/A, Brescia 25124, Italy; E-Mail: cristianosabelli@izo.it; 3 Centro Fauna Selvatica “Il Pettirosso”, Via Nonantolana 1217, Modena 41122, Italy; E-Mail: enrica.rosti@gmail.com

**Keywords:** coronavirus, bats, Italy, molecular characterization, phylogenetic analysis

## Abstract

Bats are natural reservoirs for many mammalian coronaviruses, which have received renewed interest after the discovery of the severe acute respiratory syndrome (SARS) and the Middle East respiratory syndrome (MERS) CoV in humans. This study describes the identification and molecular characterization of alphacoronaviruses and betacoronaviruses in bats in Italy, from 2010 to 2012. Sixty-nine faecal samples and 126 carcasses were tested using pan-coronavirus RT-PCR. Coronavirus RNAs were detected in seven faecal samples and nine carcasses. A phylogenetic analysis of RNA-dependent RNA polymerase sequence fragments aided in identifying two alphacoronaviruses from Kuhl’s pipistrelle (*Pipistrellus kuhlii*), three clade 2b betacoronaviruses from lesser horseshoe bats (*Rhinolophus hipposideros*), and 10 clade 2c betacoronaviruses from Kuhl’s pipistrelle, common noctule (*Nyctalus noctula*), and Savi’s pipistrelle (*Hypsugo savii*). This study fills a substantive gap in the knowledge on bat-CoV ecology in Italy, and extends the current knowledge on clade 2c betacoronaviruses with new sequences obtained from bats that have not been previously described as hosts of these viruses.

## 1. Introduction

Coronaviruses (CoVs), order *Nidovirales*, family *Coronaviridae*, subfamily *Coronavirinae*, are enveloped, single-stranded positive-sense RNA viruses with a large genome of 26 to 32 kb [1]. They are classified in four genera. *Alphacoronavirus* (*α-CoV*) and *Betacoronavirus* (*β-CoV*), which can be further grouped into clades 2a to 2d, that infect mainly mammals, and *Gammacoronavirus* (*γ-CoV*) and *Deltacoronavirus* (*δ-CoV*) that infect mainly birds [2,3]. Infection with CoVs may be asymptomatic; however, they can be responsible for a range of diseases of veterinary and medical importance, including respiratory tract infections, gastroenteritis, hepatitis, and encephalomyelitis. CoVs have a high potential for interspecies transmission. Their large genome is susceptible to mutation and recombination events, thus, new strains and viruses may originate and spread in a wide variety of animals [4,5]. 

The aetiological agent of severe acute respiratory syndrome (SARS), which originated in China and caused a global epidemic in 2003, was identified as a novel 2b *β-CoV* [6]. In 2005, the Chinese horseshoe bat (*Rhinolophus sinicus*) was recognized as the natural host of SARS-CoVs while civets and other mammals were intermediate amplifying hosts [7,8]. Since then, a growing number of novel *α-CoVs* and *β-CoVs* have been identified in bats of various species, leading to the hypothesis that bats may be the original hosts for recognized mammalian CoVs [9,10,11,12,13,14,15]. 

The most recently emerged novel human CoV is named Middle East respiratory syndrome-CoV (MERS-CoV) and likely has a bat origin, as does SARS-CoV. From September 2012 to September 2013, 52 deaths occurred among 110 laboratory-diagnosed MERS-CoV patients in the Middle East, Europe, and Africa. MERS-CoV is included in clade 2c of *β-CoV* and is most closely related to the *Pipistrellus* bat CoVs, which were identified from bats in several European countries (The Netherlands, Spain, Romania, and Ukraine) [14,16,17], to the *Tylonycteris* bat CoV HKU4, to the *Pipistrellus* bat CoV HKU5 from China [18,19,20], and to the *Nycteris* bat CoVs from Ghana [17]. Until now, no animal reservoirs or intermediate hosts have been definitively identified for MERS-CoV. A recent study suggests the widespread infection of camelids at different locations in Oman with MERS-CoV or a closely related virus [21]. 

Alpha*-CoV*s and *β-CoV*s have been identified in several European countries in recent years from bats of various species [1,13,14,16,17,22,23]; however, nothing is known about the assortment of bat-CoVs in Italy. With the exception of a previous study conducted on greater horseshoe bats (*Rhinolophus ferrumequinum*) [24], nothing is known about the role of bats as hosts and reservoirs of CoVs in northern Italy, and particularly the role of Kuhl’s pipistrelle, which is the most common bat in Italian urban areas. Therefore, this study aimed to fill a substantive gap in the knowledge of bat-CoV ecology in North Italy. 

## 2. Results and Discussion

*Sampling and Necropsy*

A total of 195 samples, 126 bat carcasses and 69 fresh droppings, were collected in Lombardia and Emilia regions, Northern Italy, during the summers of 2010, 2011, and 2012 from bats of seven species. During necropsy, no macroscopic lesions suggesting infectious diseases were observed in any of the bats examined. Most revealed dehydration and traumatic injuries, such as lacerations of the wing membrane, bone fractures, haematomas, haemoperitoneum, and haemothorax. Portions of brain, intestine, and pools of viscera (lung, heart, spleen, and liver) were collected for virological testing. A list of examined samples subdivided by host species is reported in Table 1. 

**Table 1:** List of samples analyzed.

Host	Carcasses		Faeces	
	N	%	N	%
Kuhl’s pipistrelle (*Pipistrellus kuhlii*)	68	54	28	40.7
Pipistrelle bat *(Pipistrellus* spp*.)*	48	38	30	43.5
European free-tailed bat *(Tadarida teniotis)*	2	1.6	1	1.4
Common noctule (*Nyctalus noctula*)	2	1.6	1	1.4
Savi’s pipistrelle (*Hypsugo savii*)	1	0.8	2	2.9
Common long-eared bat *(Plecotus auritus)*	3	2.4	0	-
Parti-colored bat *(Vespertilio murinus)*	2	1.6	1	1.4
Lesser horseshoe bat (*Rhinolophus hipposideros*)	0	-	6	8.7
Total samples	126	100	69	100

CoV RNA was detected in seven of 69 faecal samples (10.1%) and in nine of 126 carcasses (7.2%), belonging to four bat species. Most of the CoV-positive samples were detected from Kuhl’s pipistrelle, which was the predominantly sampled bat species in this study. CoVs were also detected from Savi’s pipistrelle (one), common noctule (one), lesser horseshoe bat (four), and Pipistrelle bat (*Pipistrellus* spp.) (one). In Table 2, there is a detailed list of samples, including the GenBank accession number assigned to the gene sequence that tested positive by RT-PCR.

**Figure 1:**
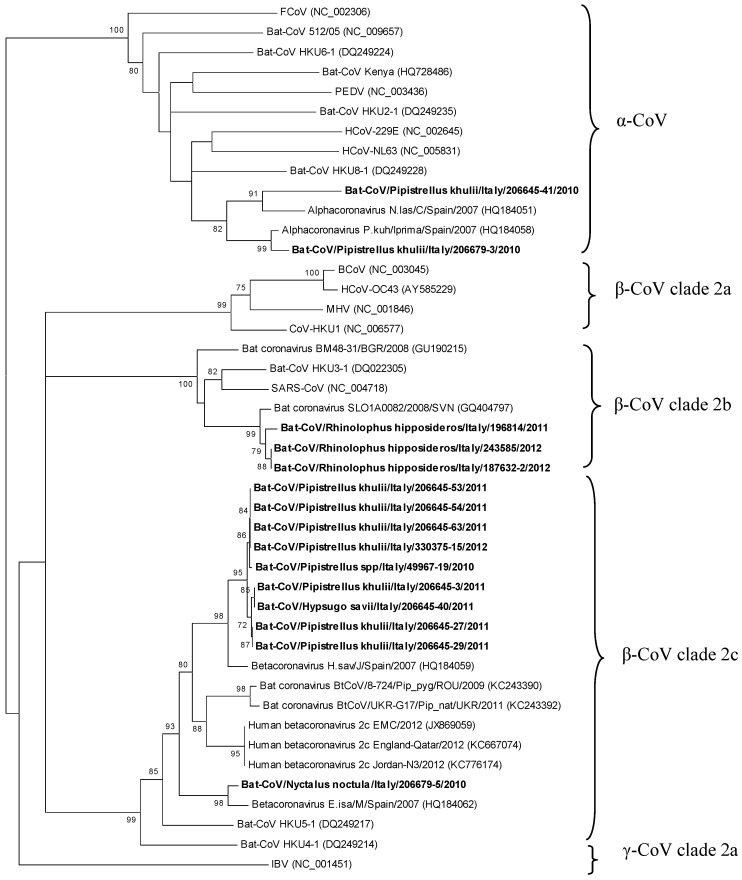
Phylogenetic tree of the partial RNA-dependent RNA polymerase (*RdRp*) gene (385 bp) of coronavirus (CoV) strains found in bats. The unrooted tree was generated by the MEGA5 program using the Neighbor-joining method. The evolutionary distances were computed using the Maximum composite likelihood model. Bootstrap values were calculated on 1,000 replicates and only values higher than 70% are shown. Coronavirus sequences detected in this study are shown in bold font.

To characterize the overall diversity of CoV sequences, a phylogenetic analysis of bat CoVs was performed using a 385 bp amplified fragment of the *RdRp* gene from 15 positive samples. For one specimen (143488/2011), it was impossible to amplify such a fragment. Nucleotide sequence analysis showed that bat CoVs belong to *α-CoV* (two samples) and to clade 2b (three samples) or 2c (10 samples) *β-CoV* (Figure 1). 

**Table 2:** List of positive samples with the GenBank accession number assigned to the gene sequences.

N ° ID	Host species	Sample type	Sex	Age	CoV strain	Group	GenBank accession number	Nucleotide similarity (%)
206679-3/2010	Kuhl’s pipistrelle	Faeces	F	Adult	Bat-CoV/Pipistrellus khulii/Italy/206679-3/2010	α	KF500949	97% Alphacoronavirus P.kuh/Iprima/Spain/2007
206679-5/2010	Common noctule	Faeces	F	Adult	Bat-CoV/Nyctalus noctula/Italy/206679-5/2010	β 2c	KF500941	96% Betacoronavirus E.isa/M/Spain/2007
206645-3/2011	Kuhl’s pipistrelle	Carcass *	M	Juvenile	Bat-CoV/Pipistrellus khulii/Italy/206645-3/2011	β 2c	KF500942	94% Betacoronavirus H.sav/J/Spain/2007
206645-27/2011	Kuhl’s pipistrelle	Carcass *	F	Adult	Bat-CoV/Pipistrellus khulii/Italy/206645-27/2011	β 2c	KF500943	94% Betacoronavirus H.sav/J/Spain/2007
206645-29/2011	Kuhl’s pipistrelle	Carcass *	F	Adult	Bat-CoV/Pipistrellus khulii/Italy/206645-29/2011	β 2c	KF500944	94% Betacoronavirus H.sav/J/Spain/2007
206645-40/2011	Hypsugo savii	Carcass *	F	Adult	Bat-CoV/Hypsugo savii/206645-40/2011	β 2c	KF500940	94% Betacoronavirus H.sav/J/Spain/2007
206645-41/2010	Kuhl’s pipistrelle	Carcass *	M	Juvenile	Bat-CoV/Pipistrellus khulii/Italy/206645-41/2010	α	KF500945	86% Alphacoronavirus N.las/C/Spain/2007
206645-53/2011	Kuhl’s pipistrelle	Carcass *	nd	nd	Bat-CoV/Pipistrellus khulii/Italy/206645-53/2011	β 2c	KF500946	94% Betacoronavirus H.sav/J/Spain/2007
206645-54/2011	Kuhl’s pipistrelle	Carcass ^	nd	nd	Bat-CoV/Pipistrellus khulii/Italy/206645-54/2011	β 2c	KF500947	94% Betacoronavirus H.sav/J/Spain/2007
206645-63/2011	Kuhl’s pipistrelle	Carcass *	nd	nd	Bat-CoV/Pipistrellus khulii/Italy/206645-63/2011	β 2c	KF500948	94% Betacoronavirus H.sav/J/Spain/2007
143488/2011	Pipistrelle bat	Faeces	nd	nd	Bat-CoV/Rhinolophus hipposideros/Italy/143488/2011	β 2b ˜	NA	
49967-19/2010	Pipistrelle bat	Carcass °	nd	Juvenile	Bat-CoV/Pipistrellus spp/Italy/49967-19/2010	β 2c	KF500951	94% Betacoronavirus H.sav/J/Spain/2007
187632-2/2012	Lesser horseshoe bat	Faeces	F	nd	Bat-CoV/Rhinolophus hipposideros/Italy/187632-2/2012	β 2b	KF500952	97% Bat coronavirus SLO1A0082/2008/SVN
243585/2012	Lesser horseshoe bat	Faeces	F	nd	Bat-CoV/Rhinolophus hipposideros/Italy/243585/2012	β 2b	KF500953	97% Bat coronavirus SLO1A0082/2008/SVN
196814/2011	Lesser horseshoe bat	Faeces	nd	nd	Bat-CoV/Rhinolophus hipposideros/Italy/196814/2011	β 2b	KF500954	97% Bat coronavirus SLO1A0082/2008/SVN
330375-15/2012	Kuhl’s pipistrelle	Faeces	M	Adult	Bat-CoV/Pipistrellus khulii/Italy/330375-15/2012	β 2c	KF500950	94% Betacoronavirus H.sav/J/Spain/2007

* CoV detected from intestine; ^ CoV detected from pool of viscera (lung, heart, spleen, and liver); ° CoV detected from both intestine and pool of viscera (lung, heart, spleen, and liver); ˜ Classified on the basis of the 180-bp RdRp gene sequence; NA, sequence not available.

Among the *α-CoV*s, the sequence of one sample, 206645-41/2010, detected from the intestine of Kuhl’s pipistrelle, was closely related (86% of nucleotide sequence similarity) to the Bat-CoV N.las/C/Spain/2007 [GenBank:HQ184051] found in a greater noctule bat (*Nyctalus lasiopterus*) in Spain, in 2007. The other *α-CoV* sequence (206679-3/2010) detected in a faecal sample from Kuhl’s pipistrelle*,* clustered with the Bat-CoV P.kuh/Iprima/Spain/2007 [GenBank:HQ184058] identified in Spain, in 2007, from the same species of bat [16]. Their reciprocal nucleotide similarity was 97%. Within the clade 2b of *β-CoV*, all three CoVs were detected in samples of bat guano (187632-2/2012, 243585/2012 and 196814/2011) collected from two reproductive colonies of lesser horseshoe bats (Table 2). The obtained sequences showed the highest nucleotide sequence similarity (97%) with the bat-CoV SLO1A0082/2008/SVN [GenBank:GQ404797] from the same bat species in Slovenia [20]. Within the clade 2c *β-CoV,* the ten CoVs were detected from faecal samples and carcasses of Kuhl’s pipistrelle (seven)*,* Savi’s pipistrelle (one), common noctule (one), and an unidentified species of the genera *Pipistrellus* (one). The sequence of the CoV from the faecal sample 206679-5/2010 of the common noctule was closely related (96% nucleotide similarity) to a *β-CoV* described in the Isabelline serotine bat (*Eptesicus isabellinus*) from Spain, in 2007, Bat-CoV E.isa/M/Spain/2007 [GenBank:HQ184062]. The remaining nine samples clustered together and were all closely related (94% nucleotide similarity) to a 2c *β-CoV* previously found in Savi’s pipistrelle in Spain, in 2007 (Bat-CoV H.sav/J/Spain/2007, [GenBank:HQ184059]) [16] (Table 2 and Figure 1). A comparison of the nucleotide and amino acid (aa) sequences of the *RdRp* fragment showed that the Italian 2c *β-CoVs* were closely related to MERS-CoV (85.2% to 87% nt identity and 95.3% to 96.1% aa identity) and that, together with the other European bat 2c *β-CoV*s, they showed a higher identity with MERS-CoV compared to the HKU4 and HKU5 Chinese strains (95.3 to 97.6% *versus* 88.2% to 92.1% aa identity). 

All the attempts to isolate bat CoVs using the cell lines were unsuccessful based on the absence of cytopathic effects (CPE); RT-PCR reactions using the 3^rd^ passage cryolysates were also negative.

## 3. Experimental

### 3.1. Sampling

Bat samples were collected from 2010 to 2012 from bats of seven species and analyzed for the presence of CoVs. The bats species were identified based on their morphologic characteristics according to the European bat identification keys [25]. The survey did not encompass any direct manipulations of bats and relied entirely on the collection of faecal samples and bat carcasses from Lombardy and Emilia Romagna, two regions in Northern Italy. These were mainly provided by rehabilitation centers in cases of bat deaths, but a limited number of samples were collected directly from known roost sites. To exclude any potential risk of rabies, brain samples were taken and examined by fluorescent antibody test prior to performing any other analyses.

### 3.2. Genome Detection and Sequencing

Faecal and organ samples were homogenized in Minimal Essential Medium (MEM, 1 g/10 mL) containing antibiotics and clarified by centrifugation at 3,000 ×*g* for 15 min. Viral RNA was extracted from 100 µL of sample using the NucleoMag 96 Virus kit (Macherey-Nagel, Düren, Germany). The RNA was eluted in 100 µL of MV6 elution buffer and stored at −80 °C. CoV screening was performed by a pan-coronavirus one-step RT-PCR method based on degenerate primers, followed by sequencing of the amplified product (180 bp) to confirm CoV identification. The pan-coronavirus primers (IZS-CoV forward 5'-CDCAYGARTTYTGYTCNCARC-3' and IZS-CoV reverse 5'-RHGGRTANGCRTCWATDGC-3') were designed by alignment of the RNA-dependent RNA polymerase (*RdRp*) gene sequences of CoVs present in the GenBank database, which allowed a broad-spectrum detection of genetically distant CoVs. An additional file shows the sequences of CoVs present in the GenBank database used for primer design (see Additional file 1). PCR was performed by adding 5 µL of extracted RNA to 20 µL of the OneStep RT-PCR kit (Qiagen, Hilden, Germany) reaction mixture containing 1 µM of each primer. RT-PCR was carried out at 50 °C for 30 min, followed by the activation of the Hot-start DNA polymerase at 95 °C for 15 min, and by 50 cycles in three steps: 94 °C for 30 s, 45 °C for 30 s, and 72 °C for 1 min. An additional extension for 10 min at 72 °C was added at the end of the run. The *RdRp* PCR products were gel purified using NucleoSpin® Gel and PCR Clean-up (Macherey-Nagel), and were subjected to nucleotide sequence analysis.

For phylogenetic analysis, a second RT-PCR was performed to amplify a 440-bp-long fragment of the highly conserved *RdRp* gene using the primers Corona 1 forward, 5'-GGTTGGGACTATCCTAAGTGTGA-3', and Corona 2 reverse, 5'-CCATCATCAGATAGAATCATCATA-3' [26]. Six samples failed to be amplified by these primers. In those cases the RT-PCR was performed using a degenerate forward primer (Corona 1 5'-GGNTGGGAYTAYCCNAARTGYGA-3') and the IZS-CoV reverse primer. The total length of the amplified fragment was 760 bp. The RT-PCR was carried out using the OneStep RT-PCR kit (Qiagen) as described above. Temperature cycling was performed as follows: 1 cycle of 50 °C for 30 min, 1 cycle of 95 °C for 15 min, 50 cycles of 30 s at 94 °C, 30 s at 48 °C, and 1 min at 72 °C, and a final elongation at 72 °C for 10 min. RT-PCR products were gel-purified and subjected to nucleotide sequence analysis. 

Cycle sequencing reactions were performed using the BigDye® Terminator Cycle Sequencing kit version 1.1 (Applied Biosystems, Foster City, CA, USA). Reactions were filtered through SigmaSpin™ Post-reaction Clean-Up Columns (Sigma, ST. Louis, MO, USA) and sequenced on an ABI PRISM 3130 Automated Capillary DNA Sequencer (Applied Biosystems) according to the manufacturer’s instructions. Both of the amplicon’s strands were sequenced with the same primers used for PCR. Nucleotide sequences were assembled with the Lasergene sequencing analysis software package (DNASTAR, Inc., Madison, WI, USA). The nucleotide sequences without the primer sequences (385 bp) were aligned and compared to 29 selected human and animal CoV sequences available from the GenBank database using ClustalW software implemented in BioEdit version 7.0.9.0. The phylogenetic trees were drawn using the Neighbor-joining method using the Maximum composite likelihood model with MEGA 5 software.

### 3.3. Virus Isolation

To isolate the coronavirus strains, CoV RNA-positive faecal and organ homogenates were inoculated in confluent monolayers of VERO cells (African green monkey kidney), MARC-145 (foetal monkey kidney), HRT-18 (human colorectal adenocarcinoma), FRhK 4 (foetal rhesus kidney), LLC-Mk2 (rhesus monkey kidney), and TB1 LU (lung, Mexican free-tailed bat, “Tadarida brasiliensis mexicana”). 

Cells were incubated at 37 °C with 5% CO_2_ and observed daily for seven days to observe the development of the CPE. In the absence of CPE, the cryolysates were sub-cultured into fresh monolayers and checked for CoVs by RT-PCR.

## 4. Conclusions

Bats are unique nocturnal mammals that possess the ability to fly. The diversity of bat species and some of their unique biological and ecological features, such as long life spans, roosting and migratory behavior, the use of torpor and hibernation, and a unique adaptive immune system, allow them to serve as reservoirs for the emergence of new viruses [27]. 

Ten years after the SARS epidemic, the recent fatal human infections caused by the novel MERS-CoV, which is closely related to Bat-CoVs recently identified in Europe, Africa and China, has resulted in a new, intense interest in the discovery of CoVs in humans and animals. Bats are increasingly recognized as natural reservoirs for mammalian CoVs and the circulation of CoVs in other mammals is the result of the occasional introduction through bats [1].

A common problem in the study of bat CoVs is the low concentration of viral RNA in faecal and organ samples that results in difficulty generating long sequences [28]. Thus, this study was limited to the analysis of a small fragment of the *RdRp* gene (385 bp). Nevertheless, it was sufficient for a phylogenetic analysis, and it was suitable to satisfy our primary goal of detecting and characterizing the coronaviruses of bats in Italy. 

Prior to this study, very little was known about the diversity of bat-CoVs, with the exception of those in a previous study in Italy, and no α*-CoV* or 2c *β-CoV* had ever been detected in bats in Italy. That study involved one species, the greater horseshoe bats, and reported a high prevalence of group 2b *β-CoV*s (SARS-like CoVs) [24]. In the present study, of the seven bat species tested, 2b *β-CoVs* were detected only from the lesser horseshoe bats. These findings reinforce and confirm a strict correlation between SARS-like CoVs and bats belonging to *Rhinolophus* spp*.* [7,22,23]. Alpha*-CoV*s were found in only two samples collected from Kuhl’s pipistrelle. This is in contrast with previous studies conduct in Germany and Spain [13,16], which showed a high prevalence of *α-CoV*s in *Pipistrellus* spp*.* bats*.*

More interestingly, clade 2c *β-CoVs* (MERS-like CoVs) in vespertilionid bats in Italy were first identified in this study. In Europe this CoV group was identified for the first time in a common pipistrelle (*Pipistrellus pipistrellus*) bat from the Netherlands, VM314 [GenBank:GQ259977] [14]. Additionally, 2c *β-CoV*s were detected in Spain from Savi’s pipistrelle BtCoVJ [GenBank:HQ184059] and Isabelline serotine bat BtCoVM [GenBank:HQ184062] [16], in Romania from Soprano pipistrelle (*Pipistrellus pigmaeus*) BtCoV8-724 [GenBank:KC243390] and in Ukraine from Nathusius’ pipistrelle (*Pipistrellus nathusii*) UKR-G17 [GenBank:KC243392] [17]. In the present study 10 novel clade 2c *β-CoV*s from Kuhl’s pipistrelle, common noctule and Savi’s pipistrelle were detected. This finding extends the current knowledge on this bat-CoV clade with new sequences obtained from bats that have not been previously described as hosts of these groups of viruses. 

The relatedness of MERS-CoV to CoVs hosted at a high prevalence by *Pipistrellus* spp. bats across Europe and the occurrence of HKU5 CoV in bats of this genus from China highlights the possibility that *Pipistrellus* spp. bats might be hosts of viruses closely related to MERS-CoV [17]. However, the genetic characterization of *Pipistrellus* spp. clade 2c *β-CoV*s and MERS-CoV revealed a marked sequence divergence in the spike and nucleocapsid proteins of HKU4 and HKU5 strains [29]. Therefore, further genetic studies are needed to better understand the evolutionary and geographic origin of MERS-CoV and the possible role of the bat as the source of the ancestral virus. However, the risk of coronavirus infection and respiratory disease in humans remains low, and bats should not be considered liabilities, especially given the vital ecosystem functions they perform [30]. In fact, in view of biodiversity preservation and the valuable ecological service that bats accomplish through their pest-controlling actions, bats are protected by national and international laws. In addition, their conservation status is often vulnerable or threatened, since these mammals are highly sensitive to anthropogenic habitat alterations. 

In conclusion, the implementation of a continuous veterinary surveillance program for detecting CoVs in bat species and in numerous countries may aid our understanding of the ecology of novel CoV infections.
